# APOE Is a Prognostic Biomarker and Correlates with Immune Infiltrates in Papillary Thyroid Carcinoma

**DOI:** 10.7150/jca.63545

**Published:** 2022-03-14

**Authors:** Xu Lin, Jing Zhang, Ru-Hua Zhao, Wen-Jing Zhang, Jing-Fang Wu, Gang Xue

**Affiliations:** 1 Department Of Histology And Embryology, Hebei North University, Zhangjiakou, 075000, China; 2 Department Of Otorhinolaryngology Head And Neck Surgery, Hebei North University, Zhangjiakou, 075000, China

**Keywords:** Papillary Thyroid Carcinoma, APOE, Immune Infiltrates, Biomarker

## Abstract

**Background:** Recent research showed that abnormal lipid metabolism was associated with cancers. As one of the genes that can regulate the level of lipid metabolism, abnormal APOE expression was associated with carcinogenesis. However, the clinical value of APOE in papillary thyroid carcinoma (PTC) remains to be determined.

**Methods:** ONCOMINE, GEPIA, UALCAN, STRING, GeneMANIA, LinkedOmics, GSCALite, TISIDB, EPIC and TIMER were utilized to achieve comprehensively bioinformatics analysis of APOE in this study. And the immunohistochemical staining of APOE was used to verify the predicted results.

**Results:** The mRNA level and protein level of APOE of PTC tissues were significantly elevated in TCGA cohort and Shanghai cohort. PTC patients with low mRNA level of APOE were associated with a bad prognosis. The functions of APOE co-expressed genes were mainly enriched in adaptive immune response, protein-lipid complex subunit organization, actin cytoskeleton reorganization, cell chemotaxis, protein activation cascade and transcriptional misregulation in cancer. APOE level was significantly correlated with tumor-infiltrating cells (B cells, CD8+ T cells, neutrophils, and dendritic) and immune biomarkers in PTC.

**Conclusions:** APOE is a potential independent biomarker for PTC and APOE expression is positively correlated with immune cell infiltration in PTC.

## Introduction

Thyroid Papillary Carcinoma (PTC) is the most common subtype of thyroid cancer, accounting for about 80% and its incidence and mortality rate have increased significantly in recent years 1, 2. As the most common endocrine tumor, PTC has a tendency to occur lymph node metastasis in the early stages of cancer, and 20%-30% of PTC patients already have cancerous lymph node involvement at the time of diagnosis 3, 4. Although some mechanisms have been clarified, including growth factors, ionizing radiation, iodine intake, gender and genetic factors, these factors have not yet specifically clarified the mechanisms of PTC occurrence and cannot be used as specific targets for clinical treatment for PTC. Meanwhile, PTC patients with lymph node metastasis have significantly higher tumor recurrence, distant metastasis and mortality rates than those without metastasis 5, 6. Thus, it is essential to identify a new biomarker that can be used to assist in screening diagnosis and predict the prognosis of PTC.

Apolipoprotein E (ApoE) can be synthesized by various tissues such as liver, intestine, adrenal gland, kidney, lung, spleen, ovary and brain. Meanwhile, APOE not only plays the role in lipid transport, storage, utilization and excretion in different organizations, it can also participate in the regulation of various diseases such as schizophrenia, coronary heart diseas, diabetic nephropathies, Alzheimer's disease and malignant tumor. Tavazoie et al pointed out that APOE may be involved in lymphocyte-mediated immune regulation, and that overexpression of APOE accelerated the biological processes of various malignant tumors 7, 8. Geng et al suggested that APOE modulated immune response by inhibiting antigen-activated lymphocytes 9. Sakashita K et al investigated the relationship between APOE and gastric cancer by immunohistochemistry and RT-PCR, and APOE overexpression in gastric cancer tissues exhibited stronger malignant invasiveness compared to cancer tissues with low APOE expression 10. And overexpression of APOE significantly promoted the abilities of invasion and lymph node metastasis of gastric cancer cells 10. There is increasing evidence that APOE is abnormally expressed in a variety of solid cancers and may be a biomarker for bladder cancer and ovarian cancer. However, the functions of APOE in PTC are still not clarified.

In this study, we analyzed the expression of APOE in PTC using Oncomine database and TCGA database, and then analyzed its clinical significance, prognostic value and immune function by various analysis tools. Our results may provide more references for further study of APOE in PTC.

## Materials and Methods

### TCGA data source and tissue samples

The data for 512 thyroid cancer tissue samples and 59 normal thyroid tissue samples were acquired from The Cancer Genome Atlas (TCGA, https://tcga-data.nci.nih.gov/tcga/ ) database. The PTC tissue microarray was bought from Shanghai Outdo Biotech CO. , LTD. Inclusion criteria: (1) All patients were diagnosed as PTC by experienced pathologists. (2) No treatment was received before surgery. Exclusion criteria: patients with incomplete information on clinicopathological data. And this study was approved by the Medical Ethics Committee of Hebei North College. Written informed consent was obtained from all participants.

### GEPIA

GEPIA (www.gepia.cancer-pku.cn/index.html) is not only a website that can quickly and easily identify the expression of specific genes in certain cancers, but also provides survival analysis, clinical information analysis and correlation analysis of specific genes. In our study, the expression analysis and prognostic analysis of APOE was performed using TCGA PTC samples with a p-value < 0.05.

### Oncomine analysis

Oncomine, an efficient data mining platform, can analyze the expression of genes in the most common cancer at the transcription level 11. In our study, the APOE expression was analyzed by Oncomine database.

### The Human Protein Atlas

The Human Pathology Atlas project (https ://www.proteinatlas.org) is a website that can help researchers to provide immunohistochemistry and immunofluorescence results of specific genes 12. In the present experiment, we compared the expression levels of APOE in papillary thyroid cancer and normal thyroid tissue.

### UALCAN analysis

The UALCAN database is a comprehensive database based on OMICS data that allows users to quickly identify gene expression, clinical information in a variety of solid tumors 13. In this study, we analyzed APOE expression by gender, age, with/without lymph node metastasis, and clinical stage with a p-value < 0.05.

### STRING and GeneMANIA

STRING (http://www.string-db.org/) is an online tool that can be used to analyze protein-protein interaction networks of multiple species 14. Similar to STRING, GeneMANIA (http://www.genemania.org) is an online database that can identify specific gene interaction networks by collecting published data in multiple databases 15. At the same time, it can also show the co-localization, co-expression and correlation between these interacting genes. After obtaining the genes from STRING, we used the GeneMANIA analysis website to make a preliminary exploration of the association and roles of these genes.

### LinkedOmics and GSCALite

LinkedOmics (www.linkedomics.org) is a complex and powerful platform that can help researchers to accurately dig up key genes from massive data by analyzing various cancer data in the TCGA database 16. GSCALite (www.bioinfo.life.hust.edu.cn/web/GSCALite/) is a powerful platform for multi-group analysis, which can directly visualize the signal pathways that may play a role in gene groups in various solid malignancies according to TCGA data 17. We extracted TCGA PTC samples and the data of 502 PTC patients were acquired. We first analyzed the genes that showed a significant correlation with APOE through the LinkFinder module. Then, the biological process, pathway, kinase, miRNA and transcription factor-target analysis of APOE was performed with a p-value < 0.05. And GSCALite provided pathway activity analysis with the TCGA PTC samples once more.

### Immune infiltration analysis

TIMER (www.cistrome.shinyapps.io/timer/), a genetic immunoassay platform, can directly perform immunological analysis on specific genes in certain cancers and visualize the results for researchers 18. At the beginning of the article, we analyzed the expression of APOE in various cancers and normal tissues through the “DiffExp module” in TIMER. Then, the correlation between APOE and the level of immune infiltrating cells in PTC and the relationship between APOE gene and biomarkers of immune cells were conducted by “gene module” and “correlation module”, respectively 19, 20. TISIDB (http://cis.hku.hk/TISIDB/) uses high-throughput analysis of genetic data to identify and predict the association of specific genes with tumor immune cell infiltration 21. To reconfirm whether APOE is involved in regulating immune infiltration in PTC, we used TISIDB to investigate the relationship between APOE expression and immunoinhibitors, immunostimulators, MHC molecules and TILs. p <0.05 was considered statistically significant. The EPIC (https://gfellerlab.shinyapps.io/EPIC_1-1/) application can help researchers to assess the proportion of immune cells and cancer cells under different expression conditions of specific genes. To evaluate the effects of APOE expression on immune cells, we divided 512 PTC samples in TCGA into two groups (APOE high expression group and APOE low expression group), and then imported the data into the EPIC application tool for analysis. Finally, the results were statistically analyzed and visualized using GraphPad Prism 7 software with p-value < 0.05.

### Immunohistochemical (IHC) staining and evaluation

Immunochemical staining of APOE was conducted using mouse monoclonal anti-APOE antibody (1: 100, catalogue number RLT0273, Ruiying Biological, China). The overall APOE IHC score grading from 1 to 5 was evaluated according to the semi-quantitative immunoreactive score (IRS) scale of Remmele 22. The results of APOE negative or positive staining in PTC were evaluated by two experienced pathologists and were determined as follows. Comprehensive immunohistochemical score = staining percentage × intensity. The staining intensities of APOE proteins were scored as follows: 0 (no staining); 1-2 (weak); >2 (strong). The positive cell percentages of APOE proteins were graded as follows: 0 (0%); 1 (1-25%); 2 (26-50%); 3 (51-75%); 4 (76-100%). APOE protein expressions were classified as follows: < 2 low expressions, ≥ 2 high expressions.

## Results

### The levels of APOE expression in THCA and other cancers

**Figure 1:**
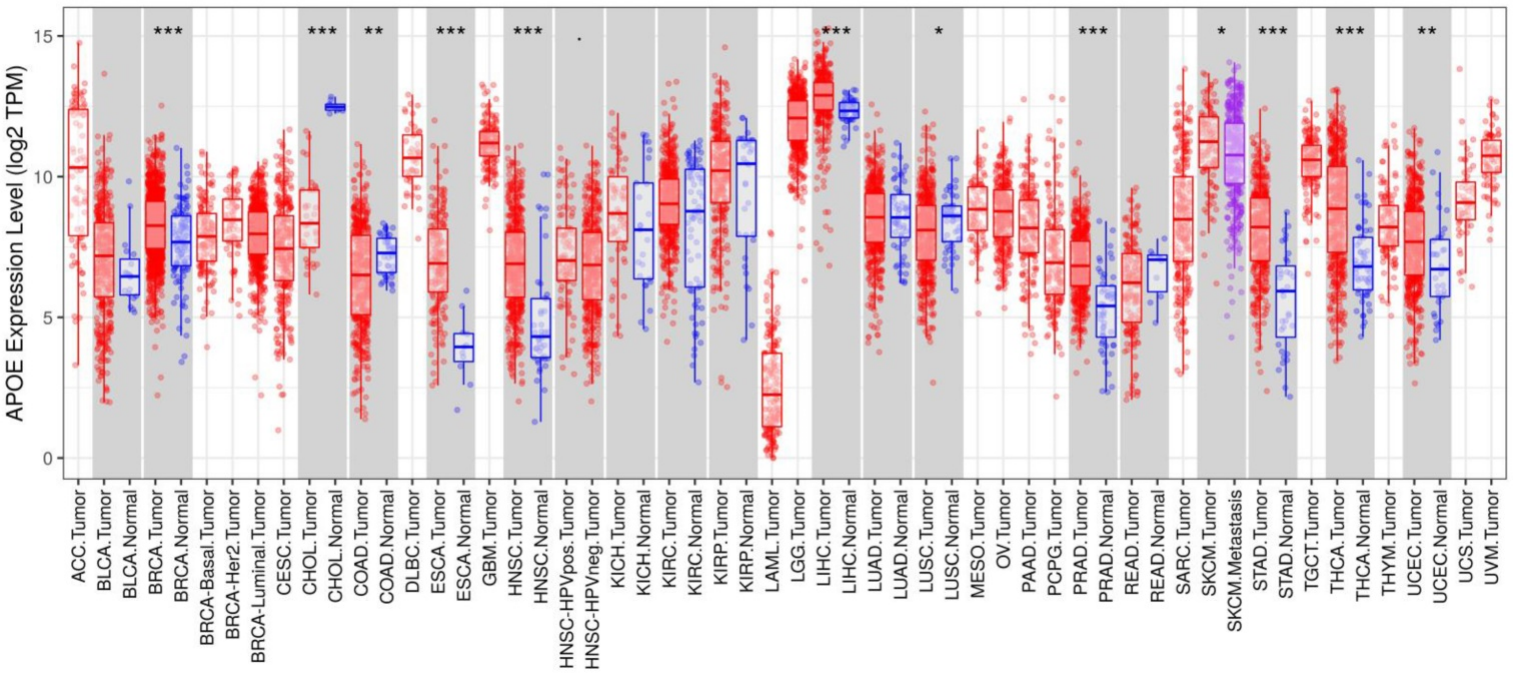
APOE expression in different types of human cancers (TIMER). Note: *P < 0.05, **P < 0.01, ***P < 0.001.

**Figure 2:**
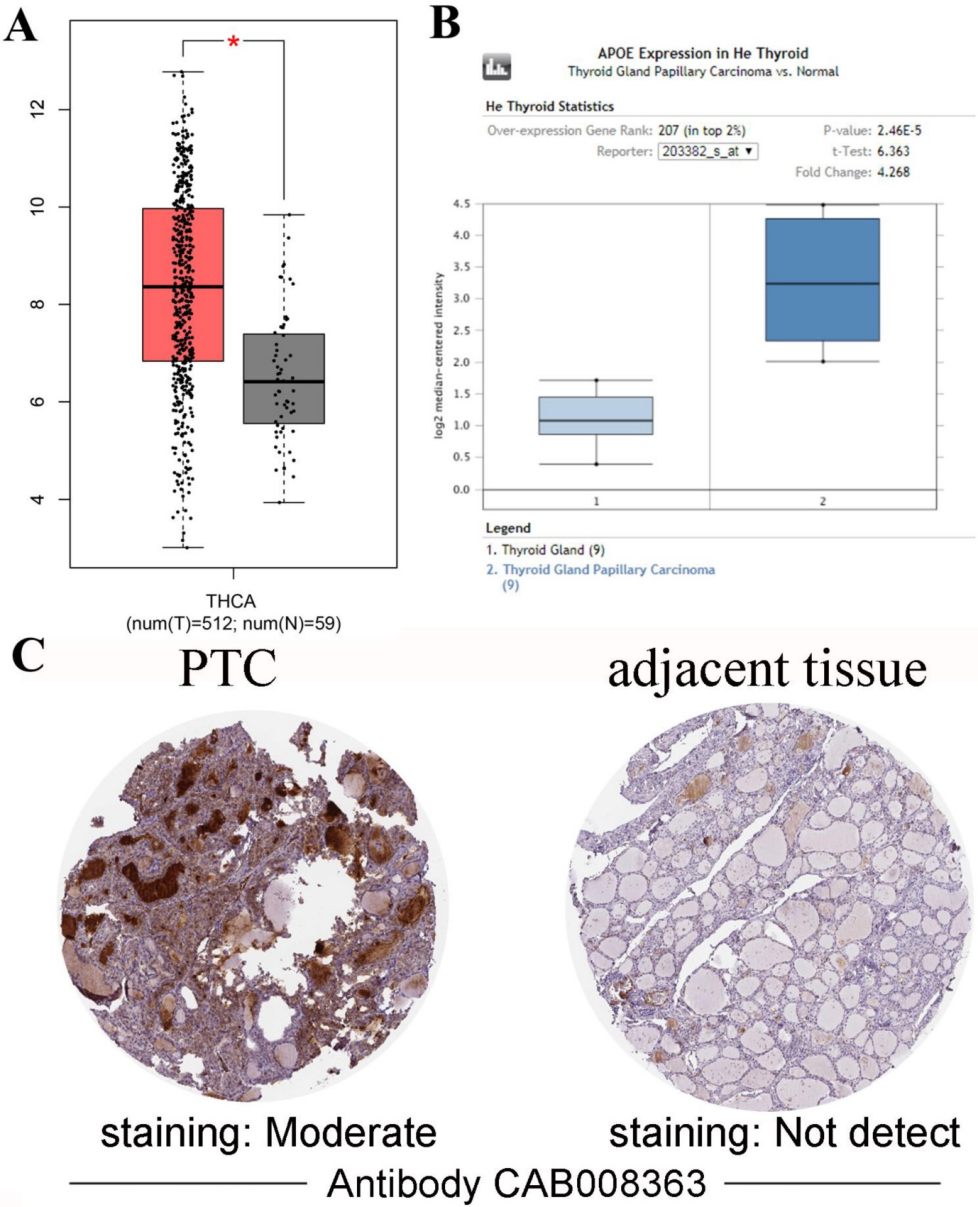
** APOE expression in PTC.** (A) Box plot comparing APOE mRNA expression in thyroid specimens from 512 patients with PTC (red) and 59 normal controls (black) from TCGA. (B) Box plot showing APOE mRNA levels in PTC specimens (dark blue) and normal controls (light blue) according to datasets from He Thyroid. (C) APOE immunohistochemical staining results of PTC and adjacent tissues according to the HPA.

Gene expression analyses using the TIMER database based on TCGA data showed that APOE mRNA levels were significantly higher in breast cancer (BRCA), Esophageal carcinoma (ESCA), Head and Neck squamous cell carcinoma (HNSC), Liver hepatocellular carcinoma (LIHC), Prostate adenocarcinoma (PRAD), Stomach adenocarcinoma (STAD), Thyroid carcinoma (THCA) and Uterine Corpus Endometrial Carcinoma (UCEC) compared with the corresponding normal tissues (Figure 1). To further investigate whether the abnormal expression of APOE affects the occurrence of thyroid cancer, we then evaluated APOE transcription levels from multi-database. As shown in Figure 2A, APOE mRNA expression was significantly upregulated in thyroid cancer than in adjacent normal tissues according to the GEPIA database. Data in the Oncomine database showed that APOE mRNA expression was significantly higher in PTC and ranked within the top 2% based on mRNA expression (Figure 2B). IHC staining according to the the Human Protein Atlas database showed that APOE expression was not detected in normal controls, while the moderate APOE expression was shown in PTC tissue (Figure 2C).

### APOE expression correlated with clinicopathological parameters for PTC patients in TCGA cohort and Shanghai cohort

**Figure 3:**
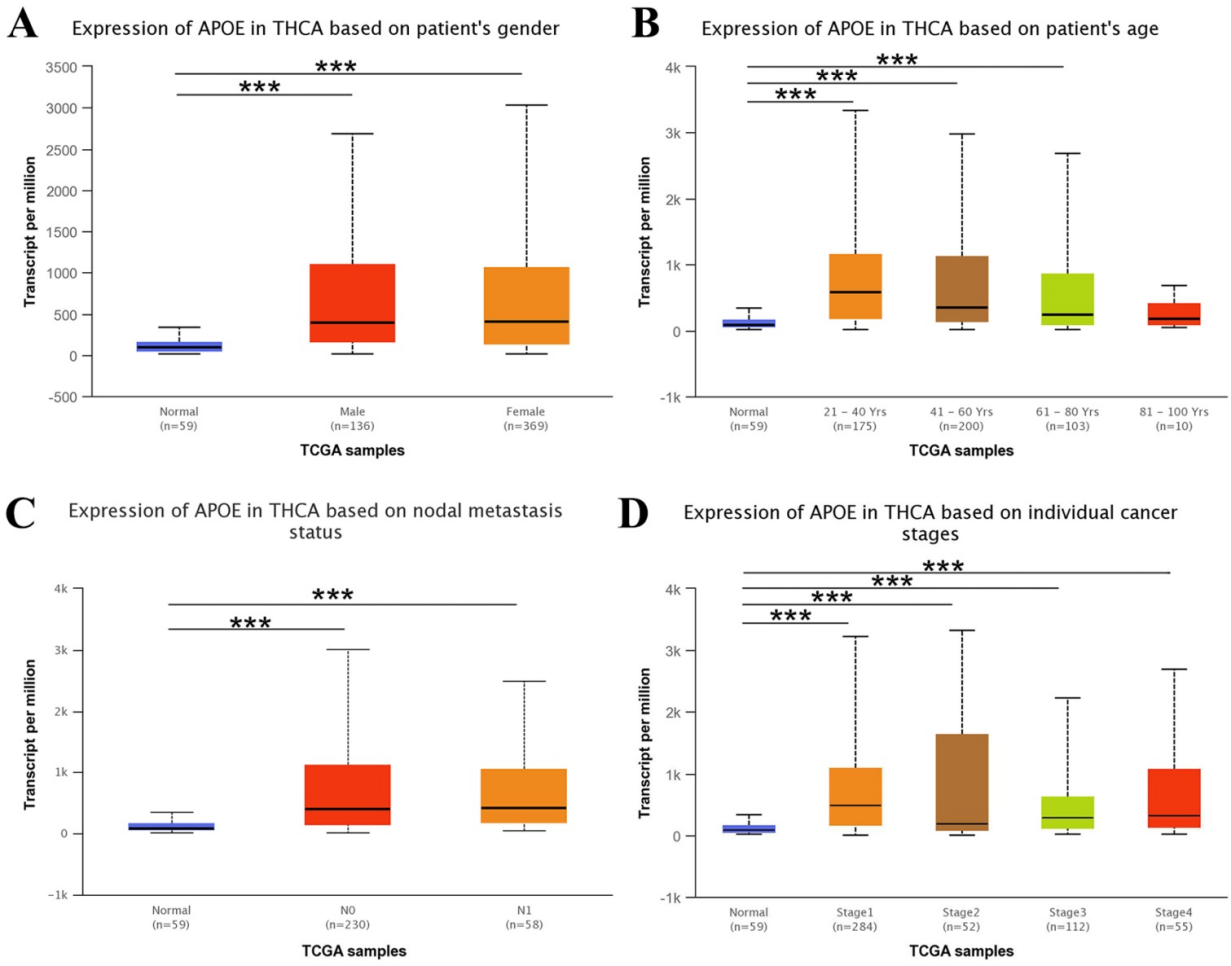
** APOE transcription in subgroups of patients with PTC.** (A) Boxplot showing relative expression of APOE based on patient's gender. (B) Boxplot showing relative expression of APOE based on patient's age. (C) Boxplot showing relative expression of APOE based on nodal metastasis status. (D) Boxplot showing relative expression of APOE in thyroid cancer based on individual cancer stages.

Next, we used the UALCAN web resource to explore the relationship between APOE expression and different clinical characteristics of thyroid cancer. As shown in Figure 3, the expression of APOE was significantly higher in PTC patients than in normal controls with multi-analysis based on gender, age, metastasis status and different stages.

**Figure 4:**
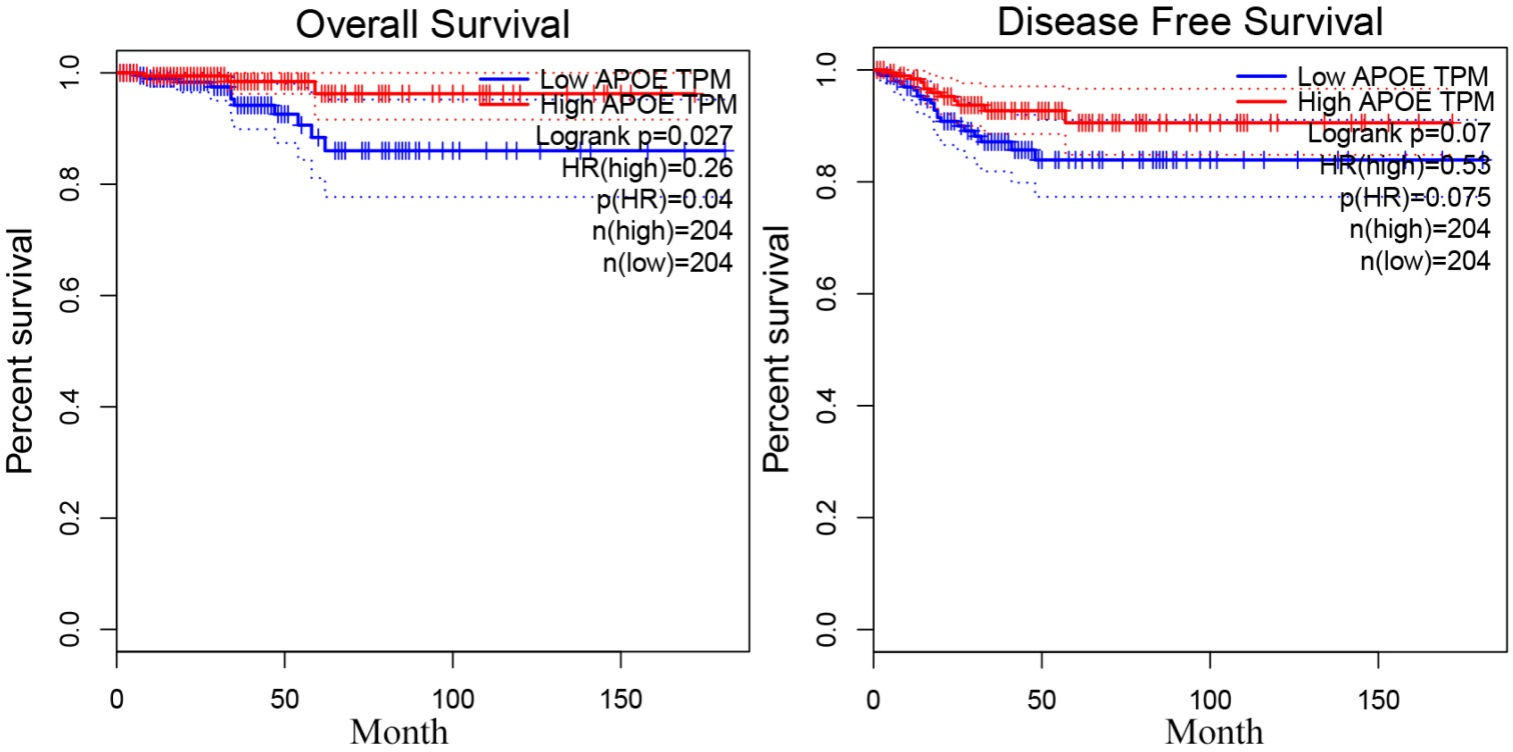
The overall survival and disease-free survival curve of APOE in PTC.

**Figure 5:**
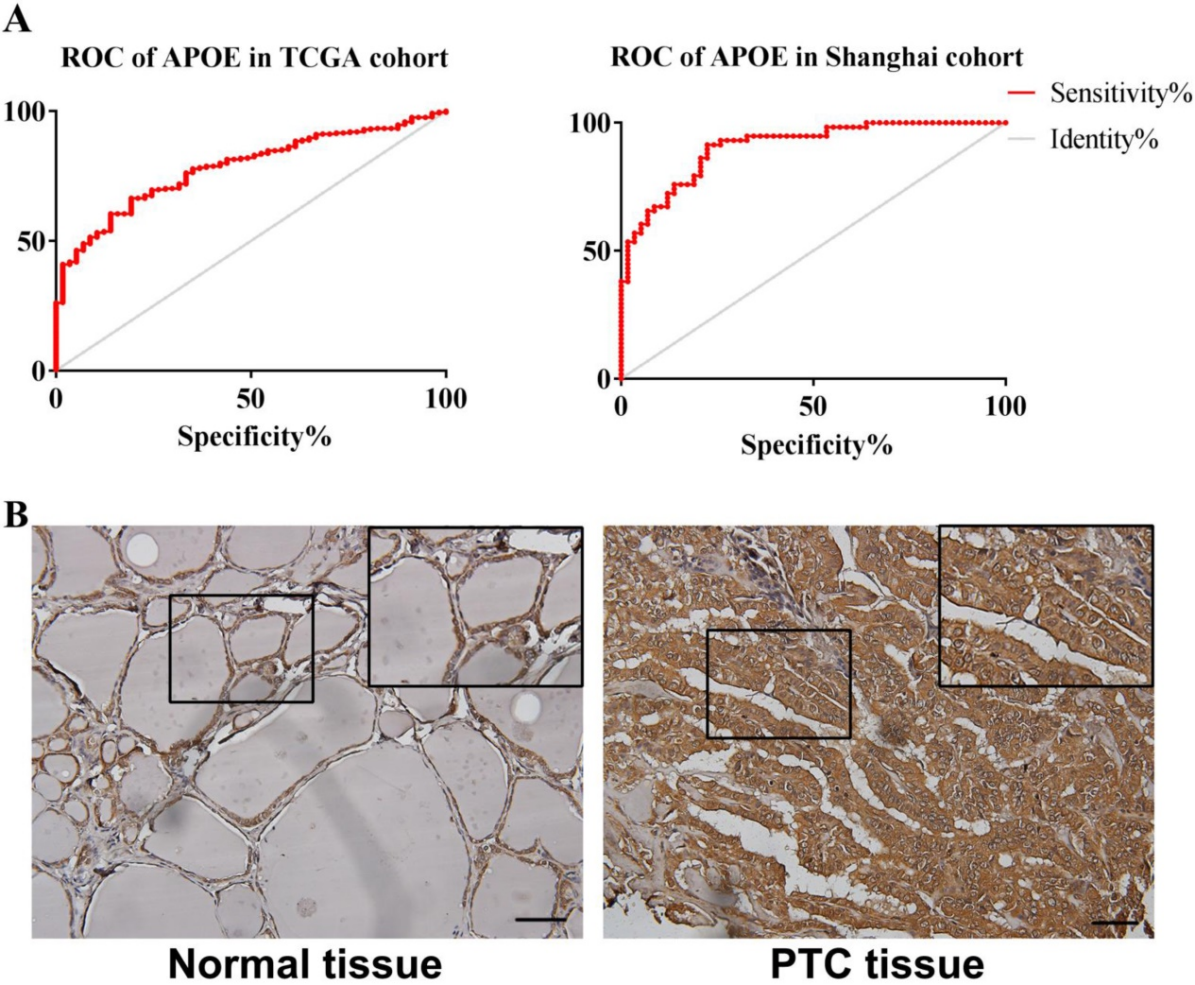
** The ROC curve and immunohistochemical staining results of APOE in TCGA cohort and Shanghai cohort.** (A) The ROC curve of TCGA cohort and Shanghai cohort. APOE in TCGA cohort , AUC=0.7819 (95%CI: 0.7314-0.8324), p < 0.0001; APOE in Shanghai cohort AUC=0.9064 (95% CI: 0.8546-0.9581), p <0.0001; (B) The immunohistochemical staining results of APOE of Shanghai cohort. APOE proteins were higher in PTC tissues, compared to tumor-adjacent tissues, bar=50μm.

In order to reveal the relationship between APOE and PTC survival outcome, the survival curve analysis based on TCGA data was analyzed by GEPIA. However, the low APOE expression group had significantly shorter overall survival (Logrank, p= 0.027) compared to the high expression group and APOE expression had no association with disease-free survival (Logrank, p = 0.07) (Figure 4). Then, matched tumor and normal samples were immunohistochemically stained to verify whether the expression of APOE protein in the Shanghai cohort was consistent with the TCGA database and The HPA database. We found that in Shanghai cohort, APOE protein expression in PTC samples was significantly increased compared with adjacent tissues: 87.6% of PTC patients had higher APOE expression levels than in the normal tissues (Figure 5). To determine the potential diagnostic value of APOE, the ROC curves of APOE were generated in TCGA cohort and Shanghai cohort, respectively (Figure 5). The ROC curve analysis showed that APOE had a satisfactory diagnostic value and the AUC of APOE were 0.7819 and 0.9064 in TCGA cohort and Shanghai cohort, respectively.

### Enrichment analysis of APOE in PTC

Two PPI networks analysis of APOE were conducted by using STRING and GeneMANIA to explore the potential interactions among the proteins interacted with APOE. 12 nodes and 778 edges were acquired in the PPI network (Figure S1A). The functions of these APOE-related genes were associated with post-translational protein modification, extracellular structure organization, inflammatory response. Results of GeneMANIA suggested that the roles of these APOE-related genes were basically linked to blood microparticle, plasma lipoprotein particle, protein-lipid complex, high-density lipoprotein particle, regulation of plasma lipoprotein particle levels, plasma lipoprotein particle remodeling and protein-lipid complex remodeling (Figure S1B).

To further understand the biological significance of APOE in PTC, LinkedOmics and GSCALite tools were used to explore the APOE co-expression patterns and possible pathways in the TCGA cohort. The results of LinkedOmics platform demonstrated 11534 genes (dark red dots) positively correlated with APOE and 8392 genes (dark green dots) negatively correlated with APOE in PTC (Figure S2A). The top 50 significant genes positively and negatively correlated with APOE in PTC were shown in Figure S2B and Figure S2C, respectively. Moreover, APOC1 (cor=0.9121, p=2.17e-195), APOC1P1 (cor=0.7111, p=2.27e-78), ISYNA1 (cor=0.6479, p=5.45e-61) and C7orf61 (cor=0.6777, p=1.21e-68) were the four hub genes most positively correlated with APOE in PTC. Enrichment analysis was also performed. GO items showed that APOE co-expressed genes mainly participated in adaptive immune response, protein-lipid complex subunit organization, artery development, actin cytoskeleton reorganization, cell chemotaxis, amyloid-beta clearance and protein activation cascade, while the activities like ER-nucleus signaling pathway, nucleoside triphosphate metabolic process, Golgi vesicle transport, nucleobase metabolic process, mitochondrial respiratory chain complex assembly, coenzyme metabolic process, mitochondrial gene expression and tricarboxylic acid metabolic process were inhibited (Figure S2D). And KEGG pathway items revealed that enrichment in transcriptional misregulation in cancer, staphylococcus aureus infection, cytokine-cytokine receptor interaction, osteoclast differentiation, neuroactive ligand-receptor interaction, allograft rejection, cholesterol metabolism, leukocyte transendothelial migration, inflammatory bowel disease (IBD), natural killer cell-mediated cytotoxicity, and ECM-receptor interaction (Figure S2D). In order to further explore the potential mechanism of the five key genes (APOC1, APOC1P1, ISYNA1, C7orf61 and APOE) and whether these genes functioned through common cancer pathways, we analyzed them using the GSCALite platform by pathway activity module. As illustrated in Figure S3, APOE participated in the activation of processes such as Apoptosis, Cell Cycle, DNA Damage Response, EMT and Hormone ER, and the inhibition of Hormone AR, PI3K/AKT, RTK and TSC/mTOR signaling pathways.

### Regulators of APOE in PTC

Owing to the significance of APOE in PTC, we further analyzed APOE networks of kinase, miRNA and transcription factor targets in PTC (Table 1). Only one kinase target of APOE was identified (Kinase_LCK) from the LinkedOmics database. Then, PPI network was constructed to reveal the underlying mechanism of kinase LCK, and showed that the function of these genes in T cell activation, regulation of lymphocyte activation / leukocyte activation, positive regulation of T cell activation / lymphocyte activation / leukocyte activation / cell activation (Figure S4). MIR-323 was enriched by GSEA for APOE co-expressed genes. Besides, V$AP1_C, V$STAT5B_01, V$NERF_Q2, V$NFKAPPAB65_01, RGAGGAARY_V$PU1_Q6, V$BACH2_01, V$NGFIC_01 and V$LBP1_Q6 were the transcription factor network targets of APOE, and the functions of these transcription factors were mostly enriched in JAK-STAT signaling pathway, MAPK signaling pathway, growth hormone receptor signaling pathway and regulation of epithelial cell migration (Figure S5).

### Association of APOE expression with immune cells and biomarkers

**Figure 6:**
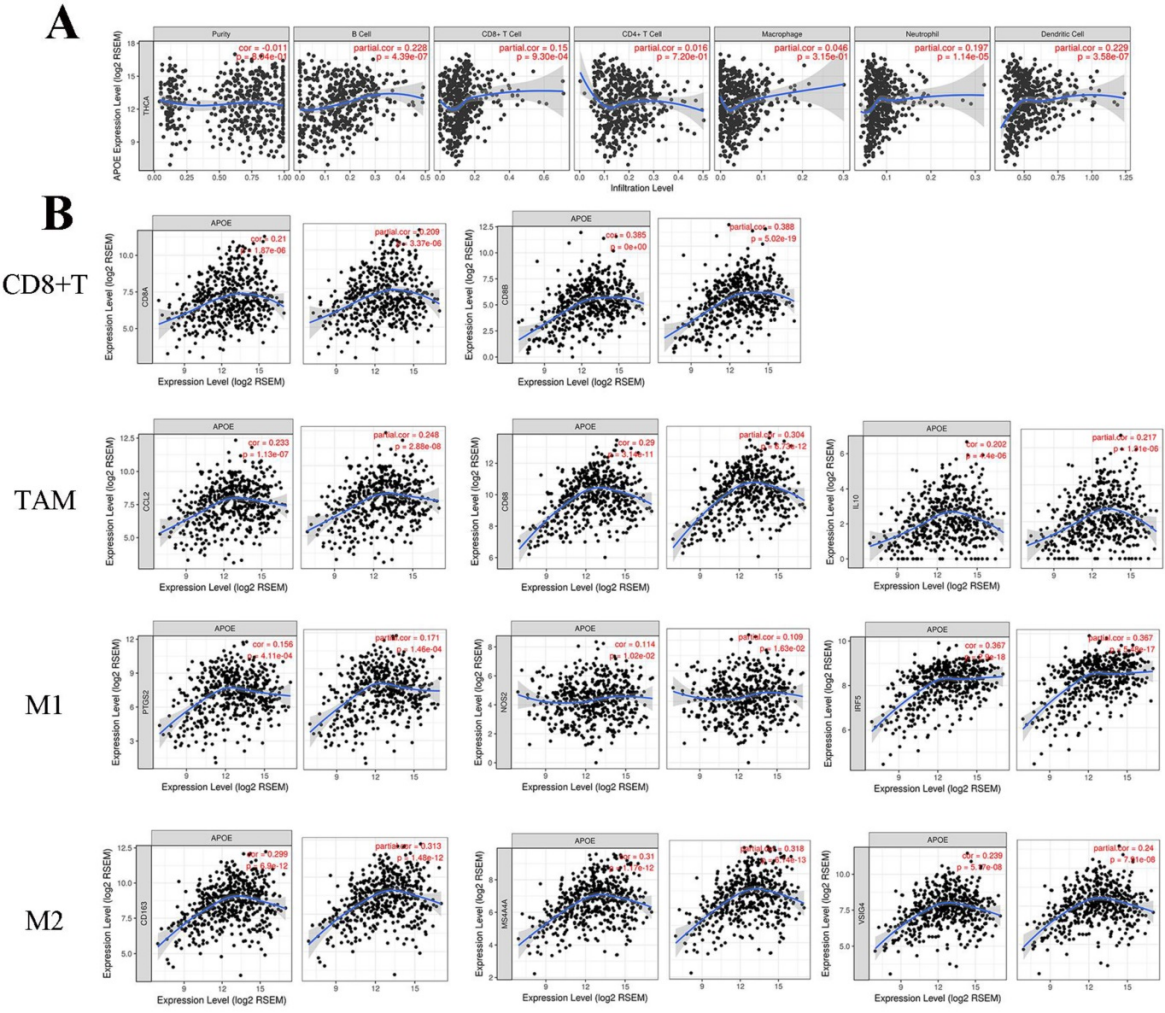
** Correlation of APOE expression with immune cell and biomarkers in thyroid cancer.** (A) APOE expression was positively correlated with B cells (Cor=0.228, P=4.39e-07), CD8+T cells(Cor=0.15, P=9.30e-4), Neutrphils (Cor=0.197, P=1.14e-05) and Dendritic cells(Cor=0.229, P=3.58e-07). (B) The APOE expression was positively correlated with immune-related markers in PTC, such as CD8+T cell, TAM, M1 and M2 macrophage.

When we analyzed the role of the APOE in PTC using the LinkedOmics platform, we found that the function of the gene was primarily focused on regulating PTC immune response. This suggested that APOE may be involved in the immunoregulatory process of PTC. Then, TIMER platform was used to further clarify the association between APOE expression and immune cell infiltration. For the correlation between APOE expression and immune-related cells, we found a positive association between APOE expression and B cells (Cor=0.228, P=4.39e-07), CD8+T cells(Cor=0.15, P=9.30e-4), Neutrphils (Cor=0.197, P=1.14e-05) and Dendritic cells(Cor=0.229, P=3.58e-07) (Figure 6A). Figure 6B showed that positive correlations were acquired between APOE expression and the expression of CD8A, CD8B. For TAM, biomarkers including CCL2, CD68 and IL10 were positively correlated with APOE expression. Similar results were obtained in M1 and M2 Macrophage (INOS (NOS2), IRF5, COX2(PTGS2), CD163, VSIG4, MS4A4A). Therefore, these results may indicate that APOE overexpression was related to the immunomodulatory process and APOE may be a potential target of immunotherapy of PTC. And multivariate COX regression analyses of APOE were performed. As shown in Table 2, tumor purity was associated with poor outcome and CD8+T cells (HR=0.000, 95%CI=0.000-0.111), macrophages (HR=0.000, 95%CI= 0.000-0.138) and Dendritic (HR=8478449.036, 95%CI=0.813-8.844267e+13) might be independent favorable prognostic indicators of PTC patients. Correlations between APOE and related gene markers of relevant immune cells were shown in Table 3.

**Figure 7:**
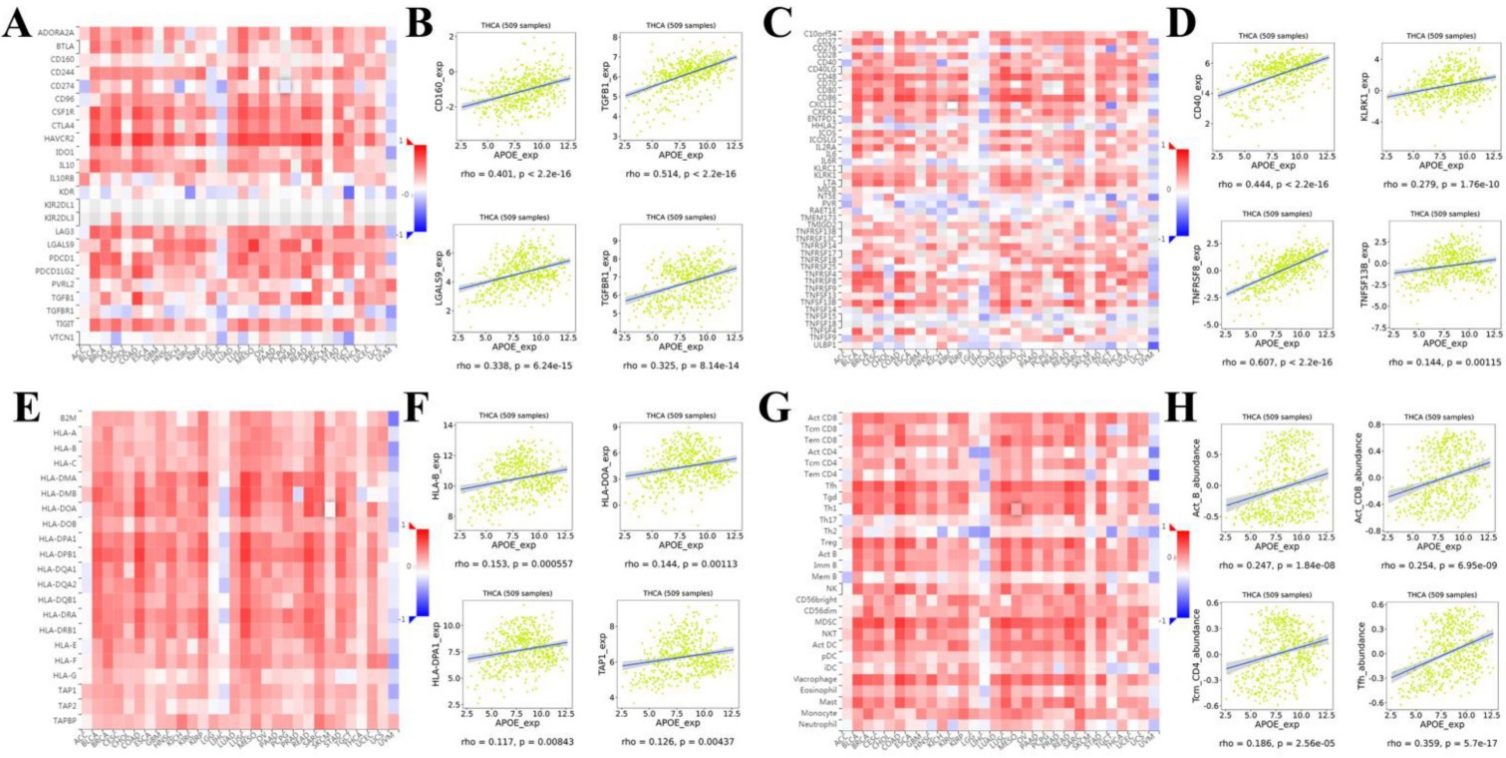
** Spearman's correlation of APOE with lymphocytes and immunomodulators (TISIDB)**. (A) Relations between the immunoinhibitors and APOE expression. (B) 4 immunoinhibitors were correlated with APOE expression. (C) Relations between the immunostimulators and APOE expression. (D) 4 immunostimulators were correlated with APOE expression. (E) Relations between the MHC molecules and APOE expression. (F) 4 MHC molecules were correlated with APOE expression. (G) Relations between the tumor-infiltrating lymphocytes and APOE expression. (H) 4 tumor-infiltrating lymphocytes were correlated with APOE expression.

In order to reveal in more detail whether there was a correlation between APOE expression and lymphocytes and immunomodulators (immunoinhibitors, immunostimulators, and major histocompatibility complex (MHC) molecules), we analyzed it using TISIDB database. Figure 7A showed correlations between APOE expression and immunoinhibitors. The immune inhibitors showed strong correlations with APOE expression including CD160 (Spearman: ρ = 0.401, P < 2.2e-16), TGFB1 (Spearman: ρ = 0.514, P < 2.2e-16), LGALS9 (Spearman: ρ = 0.338, P = 6.24e-15), and TGFBR1 (Spearman: ρ = 0.325, P = 8.14e-16) in PTC (Figure 7B). For immunostimulators, APOE expression was positively correlated with CD40 (Spearman: ρ = 0.444, P < 2.2e-16), KLRK1 (Spearman: ρ = 0.279, P = 1.76e-10), TNFRSF8 (Spearman: ρ = 0.607, P < 2.2e-16), and TNFSF13B (Spearman: ρ = 0.144, P = 0.00115) in PTC (Figure 7C-7D). Figure 7E showed correlations between APOE expression and MHC molecules. And there were positive correlations between APOE expression and HLA-B (Spearman: ρ = 0.153, P = 0.000557), HLA-DOA (Spearman: ρ = 0.144, P = 0.00113), HLA-DPA1 (Spearman: ρ = 0.117, P = 0.00843), and TAP1 (Spearman: ρ = 0.126, P = 0.00437) in PTC (Figure 7F). Besides, the correlation between APOE expression and tumor-infiltrating lymphocytes (TILs) was shown in Figure 7G, and TILs were positively correlated with APOE expression including Act_b (Spearman: ρ = 0.247, P < 1.84e-08), Act_CD8 (Spearman: ρ = 0.254, P = 6.95e-09), Tcm_CD4 (Spearman: ρ = 0.186, P = 2.56e-05), and Tfh (Spearman: ρ = 0359, P = 5.7e-17) in PTC (Figure 7H).

**Figure 8:**
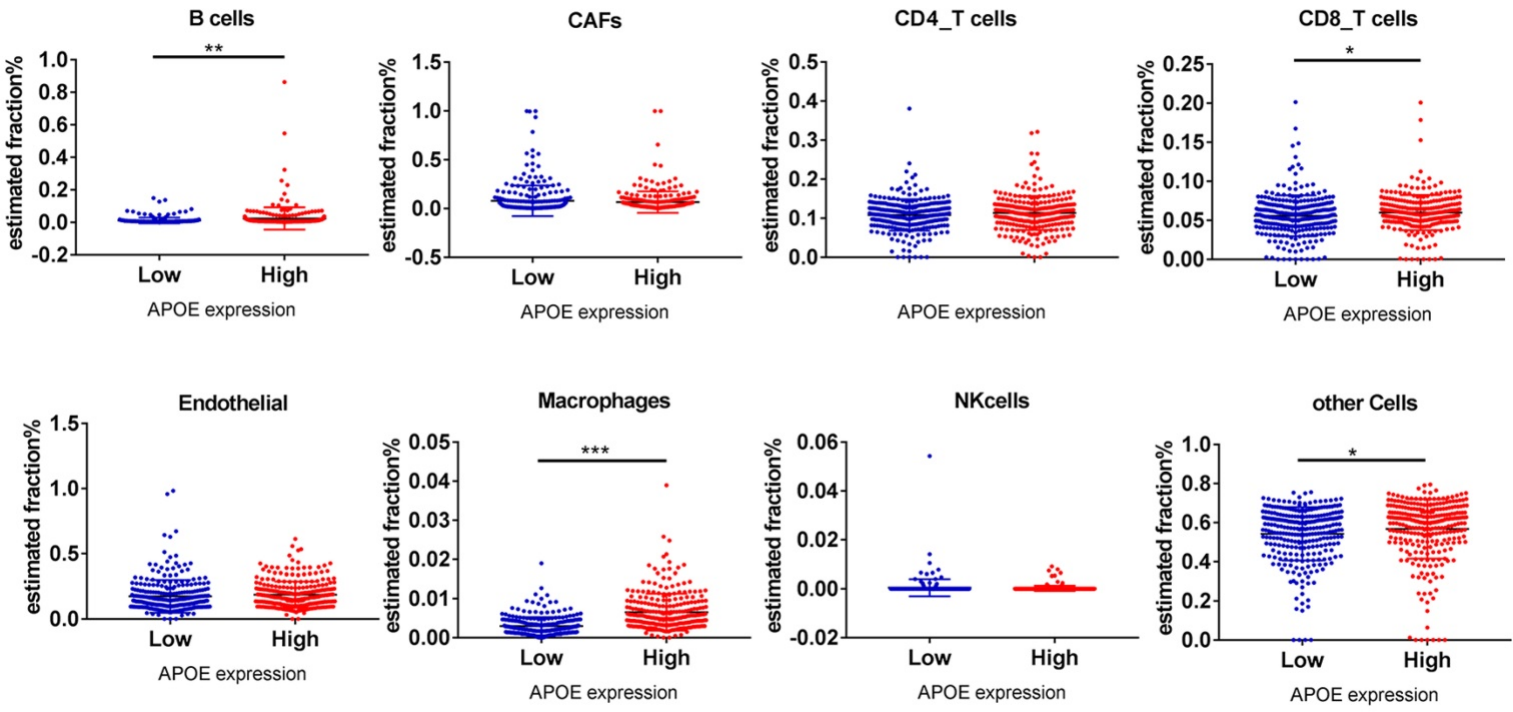
B cells, CD8_T cells, macrophages and other cells relative expression in APOE low group and APOE high group analyzing by online tools EPIC. Note: *P < 0.05, **P < 0.01, ***P < 0.001.

Finally, the EPIC application was used to analyze whether APOE expression was related to PTC immune infiltration. Among cancer samples, samples were divided into 2 groups (top 1/2 and low 1/2 APOE expression groups). As shown in Figure 8, B cells (P = 0.002), CD8 T cells (P = 0.034), Macrophage cells (P = 0.00083) and other cells (P = 0.042) were main immune cells affected by different APOE expression.

## Discussion

Previous studies had pointed out that tumor tissue needed more energy to maintain growth than normal tissue. Under this circumstance, cancer cells mainly obtained the energy necessary to maintain growth through abnormal lipid metabolism and aerobic glycolysis. More and more evidence showed that the abnormality of lipid metabolism was closely related to the occurrence of tumors 23. APOE (Apolipoprotein E), which regulated cholesterol transport and plasma protein metabolism in cells, was associated with various tumors, such as breast cancer and nervous system tumors 24, 25. At present, there were several literatures pointing out that APOE could promote the growth and metastasis of lung carcinoma and ovarian carcinoma through immunoregulatory and differentiated cell growth 26, 27. However, the function of APOE in PTC was still not clarified. In this study, we analyzed the potential immunoregulatory, cancer promoting and clinical value of APOE by bioinformatics. And the results of the bioinformatics prediction were preliminarily verified by the immunochemical staining of PTC tissue chips.

First, we extensively analyzed APOE expression in various common solid cancers through the TIMER platform. We found that APOE was more highly expressed in breast cancer, Esophageal carcinoma, head and neck squamous cell carcinoma, liver hepatocellular carcinoma, prostate adenocarcinoma, stomach adenocarcinoma, thyroid carcinoma and uterine corpus endometrial carcinoma than the corresponding adjacent tissues. Next, we detected the expression levels of APOE mRNA and protein expression level in PTC. The results from TCGA, Oncomine database and the HPA database showed that APOE mRNA and protein levels were overexpressed in PTC. And APOE mRNA levels were significantly correlated with patients' gender, age, metastasis, clinical stage and overall survival rate of PTC patients according to TCGA data. Then we performed immunohistochemical staining on the PTC tissue chip to verify whether the results were consistent with the predicted results. Some scholars pointed out that the expression level of APOE in the serum of breast cancer patients increased significantly, and these patients were more prone to metastasis 28. And consistent with the results of Xu et al, we found that APOE can also be considered as a prognostic risk factor of PTC 28. Therefore, APOE may play a potential role in PTC.

We then explored top-50genes, which may interact with APOE, and analyzed their potential roles in a variety of ways. The results of functional enrichment analysis indicated that the functions of APOE and APOE-related genes are mainly involved in post-translational protein modification, adaptive immune response, extracellular structure organization, inflammatory response, blood microparticle, plasma lipoprotein particle, protein-lipid complex, high-density lipoprotein particle, regulation of plasma lipoprotein particle levels, plasma lipoprotein particle remodeling, protein-lipid complex remodeling, protein-lipid complex subunit organization, artery development, actin cytoskeleton reorganization, cell chemotaxis, amyloid-beta clearance and protein activation cascade. And KEGG pathway items revealed that APOE was involved in transcriptional misregulation in cancer, activation of multiple signaling pathways processes such as Apoptosis, Cell Cycle, DNA Damage Response, EMT and Hormone ER, and the inhibition of Hormone AR, PI3K/AKT, RTK and TSC/mTOR signaling pathways. Chen et al pointed out that APOE mRNA expression increased more than 10-fold in ovarian cancer, and that APOE knockdown in OVCAR3 cells could lead to arrested cell growth and induce cell apoptosis 27. Previous studies had shown that elevated cholesterol levels can increase the risk of cancer. Consistent with our findings, Ben pointed out that especially in breast cancer, APOE can inhibit the proliferation and migration of tumor cells by regulating cholesterol metabolism, and APOE can be used as a target to improve prognosis 29. Moreover, APOE can regulate cancer progression by regulating lipid metabolism via TGFβ, EMT, ER signaling pathways 29, 30. Therefore, these results indicated that the function of the APOE may be related to cancer-related pathways, and APOE may mediate the occurrence of PTC.

Kinases, especially tyrosine Kinase, played an important role in regulating cell differentiation and growth under physiological conditions 31. APOE expression can be regulated by miRNA and transcription factors through its own unique regulatory mechanisms 32, 33. In our experiment, we found that the kinase and miRNA that regulate APOE were LCK, which belong to Src Family Tyrosine Kinase (PKTs) and miRNA-323, respectively. And the functions of Kinases, miRNAs and transcription factors that regulated APOE were mainly enriched in T cell activation, regulation of lymphocyte activation/leukocyte activation, positive regulation of T cell activation/lymphocyte activation/leukocyte activation/cell activation, JAK-STAT signaling pathway, MAPK signaling pathway, growth hormone receptor signaling pathway and regulation of epithelial cell migration. These results indicated that APOE may affect immune regulation through multiple signaling pathways to promote tumorigenesis of PTC via these three regulators.

Accumulating evidence indicated that APOE can participate in the immune regulation of various diseases 7, 8, 34, 35. Tavazoie et al pointed out that APOE can regulate immunity through myeloid-derived suppressor cells (MDSC) accumulation in cancers 7. Moreover, APOE-related signalings in cancers can participate in regulation of immunity 7. In this study, the correlation between APOE level and immune infiltration cells were analyzed, which suggested that high APOE was associated with immune infiltration and we found a positive association between the expression of APOE and the infiltration of B cells, CD8+T cells, Neutrophils, Dendritic cells. Currently, immunotherapy has become one of the most promising strategies in cancer treatment. In this experiment, immunoinhibitors, immunostimulators of APOE were analyzed. The results showed that CD160, TGFB1, LGALS9, TGFBR1, CD40, KLRK1, and TNFRSF8 may serve as APOE immunomodulatory targets. Thus, APOE can not only be used as a diagnostic factor for PTC, but also as a potential target for immunotherapy.

In summary, our results suggest that APOE is a potential independent biomarker for PTC and APOE expression is positively correlated with immune cell infiltration in PTC.

## Supplementary Material

Supplementary figures.

## Figures and Tables

**Table 1:** The Kinase, miRNA and transcription factor-target networks of APOE in Thyroid carcinoma (LinkedOmics).

Enriched Category	Geneset	LeadingEdgeNum	FDR
Kinase Target	Kinase_LCK	43	0.042960
miRNA Target	TAATGTG,MIR-323	56	0.017515
Transcription Factor Target	V$AP1_C	84	0.010616
	V$STAT5B_01	75	0.014154
	V$NERF_Q2	79	0.014154
	V$NFKAPPAB65_01	95	0.015974
	RGAGGAARY_V$PU1_Q6	145	0.01607
	V$BACH2_01	73	0.016728
	V$NGFIC_01	57	0.018137
	V$LBP1_Q6	53	0.01859

**Table 2:** Multivariate analyses of APOE expressions and immune cells related to overall survival in PTC.

	coef	HR	95%CI_l	95%CI_u	p.value
Age	0.213	1.238	1.119	1.369000e+00	**0.000**
gendermale	0.155	1.168	0.264	5.173000e+00	0.838
raceBlack	16.500	14650401.817	0.000	Inf	0.999
raceWhite	15.525	5524187.430	0.000	Inf	0.999
Purity	6.069	432.136	11.001	1.697427e+04	**0.001**
B_cell	0.852	2.344	0.000	4.079308e+05	0.890
CD8_Tcell	-20.499	0.000	0.000	1.110000e-01	**0.028**
CD4_Tcell	9.396	12044.886	0.240	6.037987e+08	0.089
Macrophage	-32.041	0.000	0.000	1.380000e-01	**0.037**
Neutrophil	-50.757	0.000	0.000	1.296886e+08	0.152
Dendritic	15.953	8478449.036	0.813	8.844267e+13	**0.043**
APOE	-0.393	0.675	0.442	1.030000e+00	0.069

**Table 3:** Correlation between APOE and related gene markers of relevant immune cells.

Immune cell	Gene Markers	None		Purity	
		r	p	r	p
CD8+T cell	CD8A	0.21	1.87e-06	0.209	3.37e-06
	CD8B	0.385	0E+00	0.388	5.02-19
TAM	CCL2	0.233	1.13e-07	0.248	2.88e-08
	CD68	0.29	3.14e-11	0.304	6.73e-12
	IL10	0.202	4.4e-06	0.217	1.31e-06
M1 Macrophage	INOS(NOS2)	0.114	1.02e-02	0.109	1.63e-02
	IRF5	0.367	2.92e-18	0.367	5.48e-17
	COX2(PTGS2)	0.156	4.11e-04	0.171	1.46e-04
M2 Macrophage	CD163	0.299	6.9e-12	0.313	1.48e-12
	VSIG4	0.239	5.17-08	0.24	7.91e08
	MS4A4A	0.31	1.17e-12	0.318	6.14e-13

## References

[1] James BC, Mitchell JM, Jeon HD (2018). An update in international trends in incidence rates of thyroid cancer, 1973-2007. Cancer Causes Control.

[2] Cabanillas ME, Mcfadden DG, Durante C (2016). Thyroid cancer. Lancet.

[3] Iorio MV, Croce CM (2012). MicroRNA dysregulation in cancer: diagnostics, monitoring and therapeutics. A comprehensive review. EMBO Mol Med.

[4] Haugen BR (2017). 2015 American Thyroid Association Management Guidelines for Adult Patients with Thyroid Nodules and Differentiated Thyroid Cancer: What is new and what has changed?. Cancer.

[5] Pourseirafi S, Shishehgar M, Ashraf MJ (2018). Papillary Carcinoma of Thyroid with Nasal Cavity Metastases: A Case Report. Iran J Med Sci.

[6] Ullmann TM, Gray KD, Moore MD (2018). Current controversies and future directions in the diagnosis and management of differentiated thyroid cancers. Gland Surg.

[7] Tavazoie MF, Pollack I, Tanqueco R (2018). LXR/ApoE Activation Restricts Innate Immune Suppression in Cancer. Cell.

[8] Shi Y, Holtzman DM (2018). Interplay between innate immunity and Alzheimer disease: APOE and TREM2 in the spotlight. Nat Rev Immunol.

[9] Geng H, Law PP, Ng MC (2011). APOE genotype-function relationship: evidence of -491 A/T promoter polymorphism modifying transcription control but not type 2 diabetes risk. PLoS One.

[10] Sakashita K, Tanaka F, Zhang X (2008). Clinical significance of ApoE expression in human gastric cancer. Oncol Rep.

[11] Rhodes DR, Yu J, Shanker K (2004). ONCOMINE: a cancer microarray database and integrated data-mining platform. Neoplasia.

[12] Asplund A, Edqvist PH, Schwenk JM (2012). Antibodies for profiling the human proteome-The Human Protein Atlas as a resource for cancer research. Proteomics.

[13] Chandrashekar DS, Bashel B, Balasubramanya SAH (2017). UALCAN: A Portal for Facilitating Tumor Subgroup Gene Expression and Survival Analyses. Neoplasia.

[14] Szklarczyk D, Gable AL, Lyon D (2019). STRING v11: protein-protein association networks with increased coverage, supporting functional discovery in genome-wide experimental datasets. Nucleic Acids Res.

[15] Warde-Farley D, Donaldson SL, Comes O (2010). The GeneMANIA prediction server: biological network integration for gene prioritization and predicting gene function. Nucleic Acids Res.

[16] Vasaikar SV, Straub P, Wang J (2018). LinkedOmics: analyzing multi-omics data within and across 32 cancer types. Nucleic Acids Res.

[17] Liu CJ, Hu FF, Xia MX (2018). GSCALite: a web server for gene set cancer analysis. Bioinformatics.

[18] Li T, Fan J, Wang B (2017). TIMER: A Web Server for Comprehensive Analysis of Tumor-Infiltrating Immune Cells. Cancer Res.

[19] Danaher P, Warren S, Dennis L (2017). Gene expression markers of Tumor Infiltrating Leukocytes. J Immunother Cancer.

[20] Sousa S, Maatta J (2016). The role of tumour-associated macrophages in bone metastasis. J Bone Oncol.

[21] Ru B, Wong CN, Tong Y (2019). TISIDB: an integrated repository portal for tumor-immune system interactions. Bioinformatics.

[22] Remmele W, Stegner HE (1987). [Recommendation for uniform definition of an immunoreactive score (IRS) for immunohistochemical estrogen receptor detection (ER-ICA) in breast cancer tissue]. Pathologe.

[23] Chan AW, Gill RS, Schiller D (2014). Potential role of metabolomics in diagnosis and surveillance of gastric cancer. World J Gastroenterol.

[24] Koleck TA, Bender CM, Sereika SM (2014). Apolipoprotein E genotype and cognitive function in postmenopausal women with early-stage breast cancer. Oncol Nurs Forum.

[25] Calabuig-Navarro MV, Jackson KG, Walden CM (2014). Apolipoprotein E genotype has a modest impact on the postprandial plasma response to meals of varying fat composition in healthy men in a randomized controlled trial. J Nutr.

[26] Su WP, Chen YT, Lai WW (2011). Apolipoprotein E expression promotes lung adenocarcinoma proliferation and migration and as a potential survival marker in lung cancer. Lung Cancer.

[27] Chen YC, Pohl G, Wang TL (2005). Apolipoprotein E is required for cell proliferation and survival in ovarian cancer. Cancer Res.

[28] Xu X, Wan J, Yuan L (2016). Serum levels of apolipoprotein E correlates with disease progression and poor prognosis in breast cancer. Tumour Biol.

[29] Ben Hassen C, Gutierrez-Pajares JL, Guimaraes C (2020). Apolipoprotein-mediated regulation of lipid metabolism induces distinctive effects in different types of breast cancer cells. Breast Cancer Res.

[30] Bouris P, Skandalis SS, Piperigkou Z (2015). Estrogen receptor alpha mediates epithelial to mesenchymal transition, expression of specific matrix effectors and functional properties of breast cancer cells. Matrix Biol.

[31] Jiao Q, Bi L, Ren Y (2018). Advances in studies of tyrosine kinase inhibitors and their acquired resistance. Mol Cancer.

[32] Lu TX, Rothenberg ME (2018). MicroRNA. J Allergy Clin Immunol.

[33] Lambert SA, Jolma A, Campitelli LF (2018). The Human Transcription Factors. Cell.

[34] Krasemann S, Madore C, Cialic R (2017). The TREM2-APOE Pathway Drives the Transcriptional Phenotype of Dysfunctional Microglia in Neurodegenerative Diseases. Immunity.

[35] Nam KN, Wolfe CM, Fitz NF (2018). Integrated approach reveals diet, APOE genotype and sex affect immune response in APP mice. Biochim Biophys Acta Mol Basis Dis.

