# Supplementary material for: APOE Is a Prognostic Biomarker and Correlates with Immune Infiltrates in Papillary Thyroid Carcinoma
Source: J Cancer. 2022 Mar 14;13(5):1652–63. doi: 10.7150/jca.63545 (PMC8965124; doi:10.7150/jca.63545)
Supplement: Supplementary file 1 — Supplementary figures. [file jcav13p1652s1.pdf]

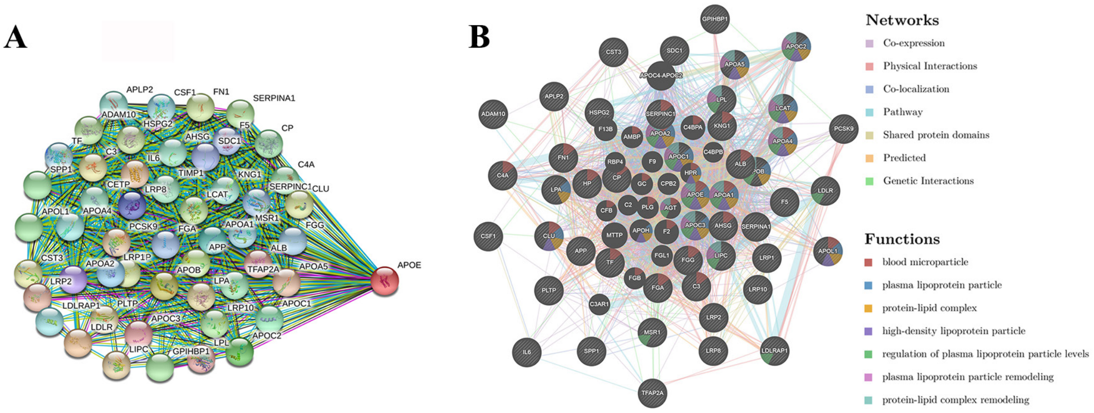

Figure S1. Neighbor gene network of differently expressed APOE in PTC patients. (A) Neighbor gene network of APOE of STRING. (B) Protein–protein interaction network of APOE in GeneMANIA.

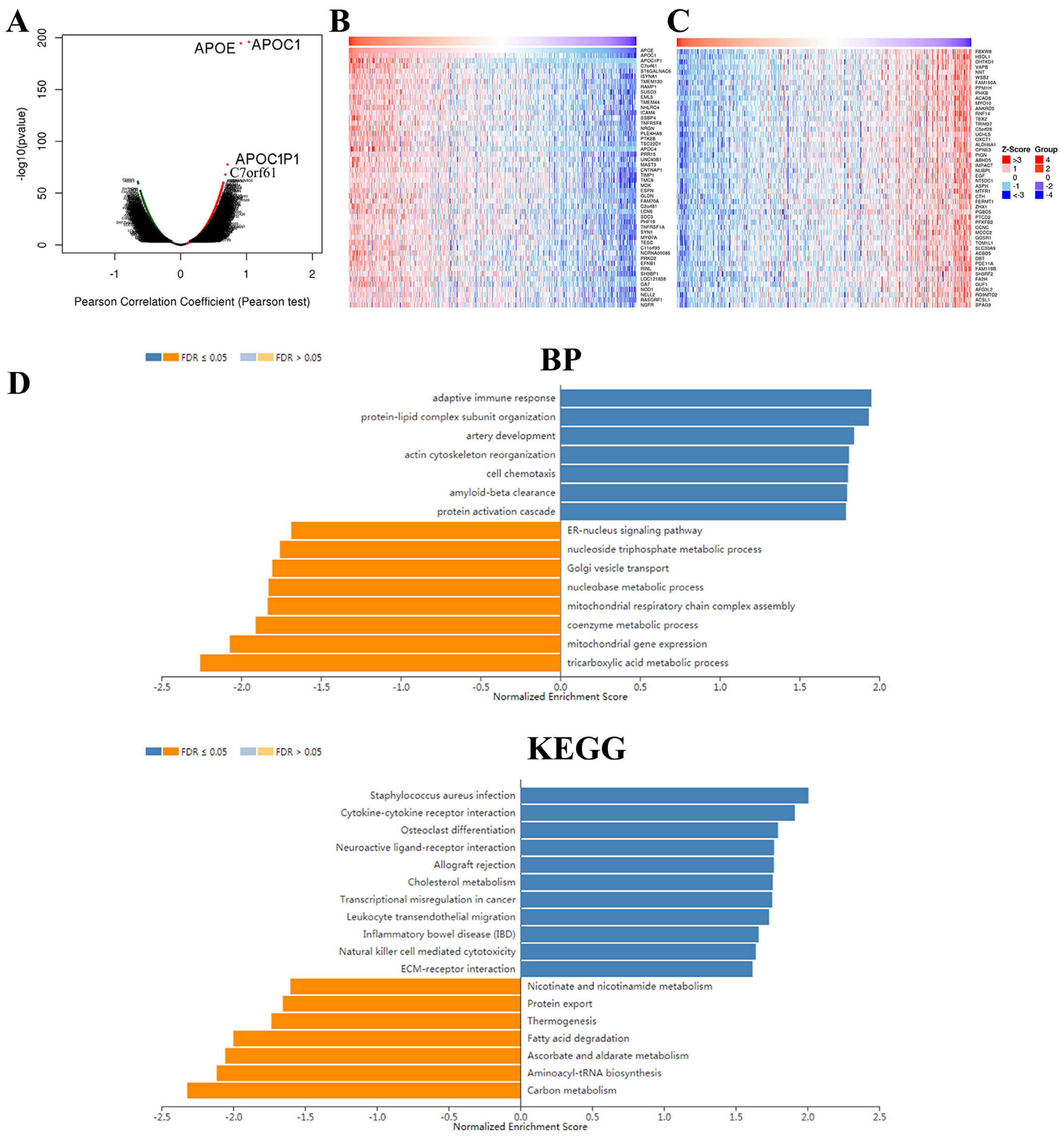

Figure S2. APOE co-expression genes in PTC (LinkedOmics). (A) The genes positively and negatively correlated with APOE in PTC. (B) The top-50 gene positively correlated with APOE in PTC in heat maps. (C) The top-50 gene negatively correlated with APOE in PTC in heat maps. (D) Biological process analyse and KEGG pathways of APOE in TCGA cohort.

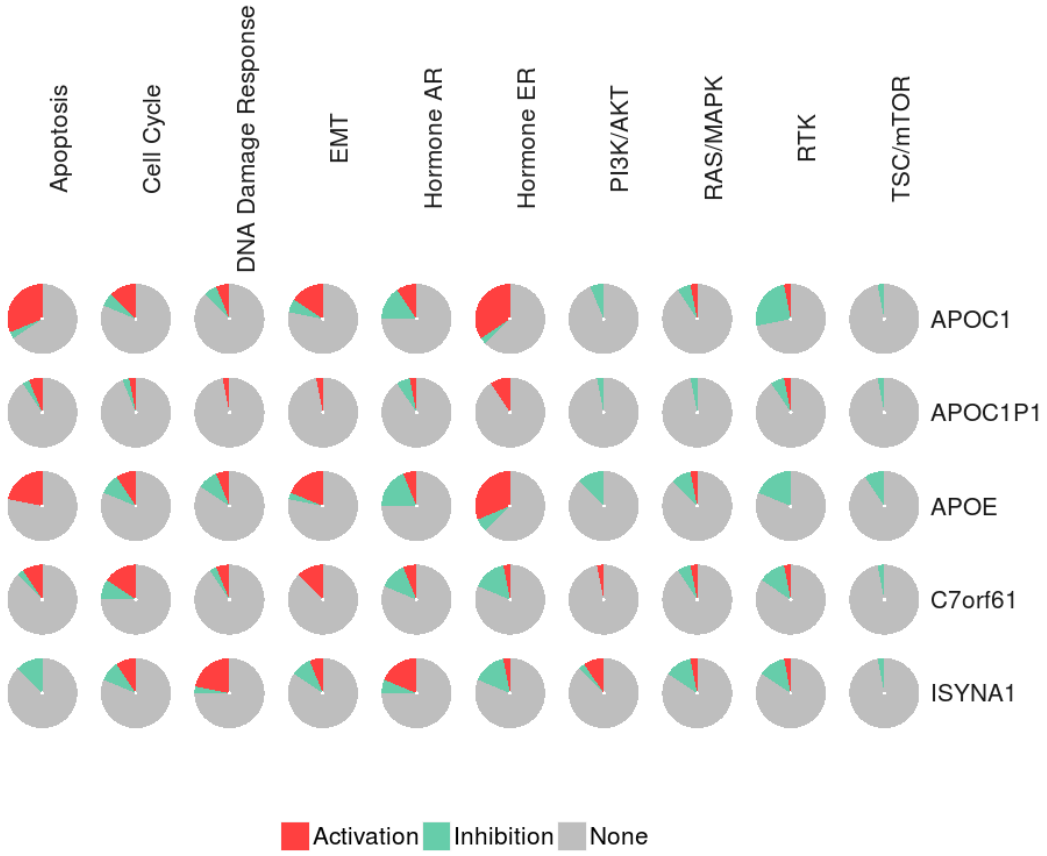

Figure S3. The role of APOE in the cancer related pathways (GSCALite).

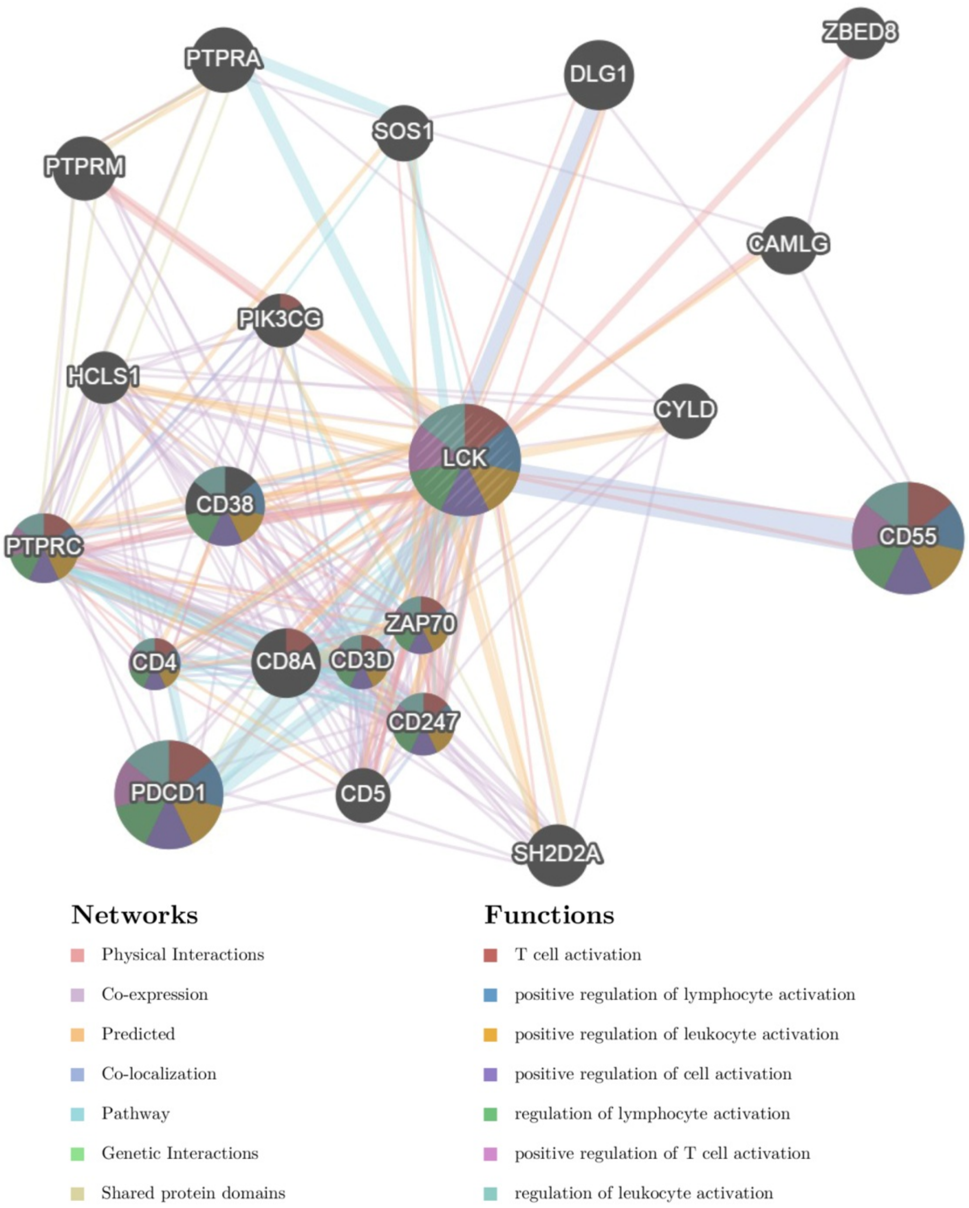

Figure S4. PPI network of kinase LCK -target networks (GeneMANIA).

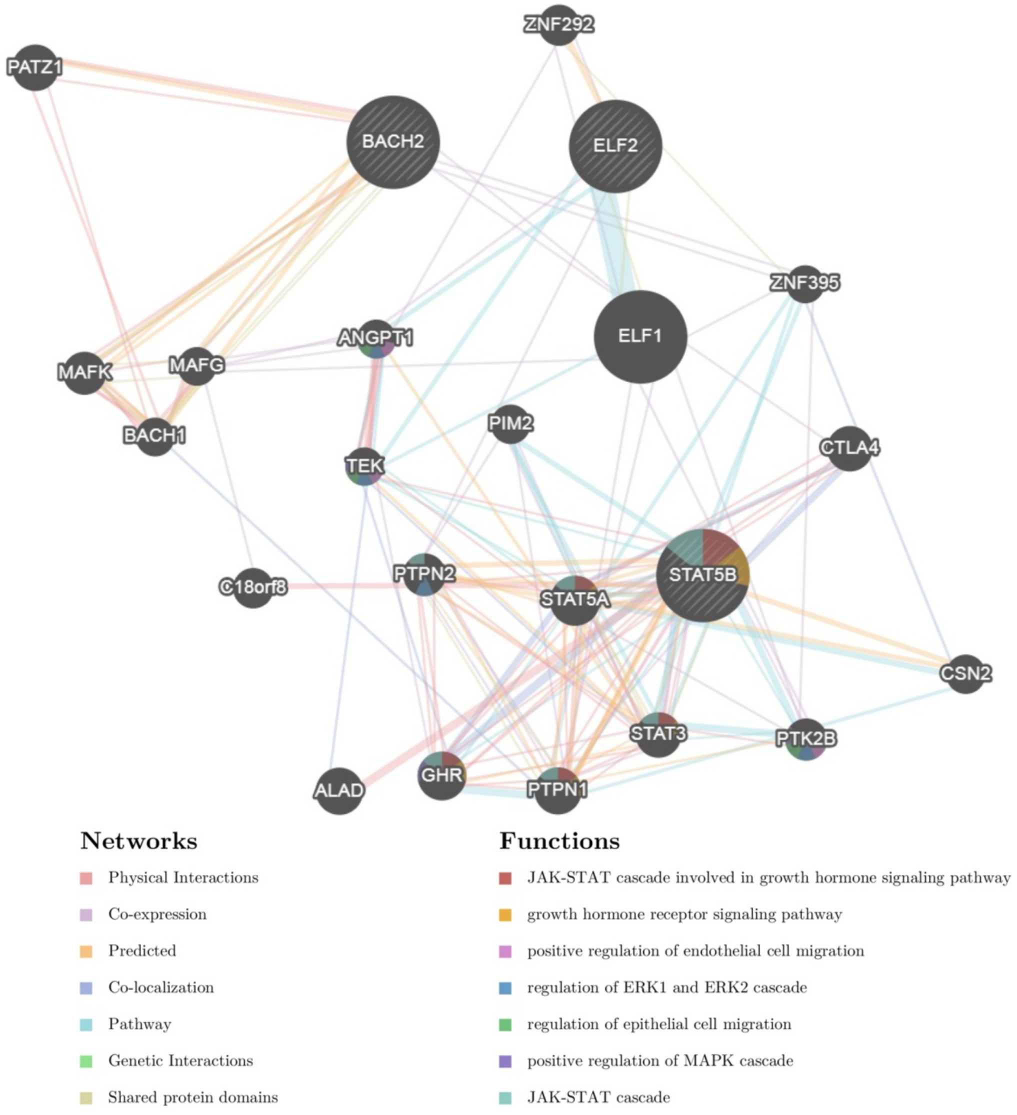

Figure S5. PPI network of transcription factors of APOE target networks (GeneMANIA).
